# Numerical Solution of Boundary Layer MHD Flow with Viscous Dissipation

**DOI:** 10.1155/2014/756498

**Published:** 2014-01-29

**Authors:** S. R. Mishra, S. Jena

**Affiliations:** ^1^Department of Mathematics, Institute of Technical Education and Research, Siksha ‘O' Anusandhan University, Khandagiri, Bhubaneswar, Odisha 751030, India; ^2^Department of Mathematics, Centurion University of Technology and Management, Bhubaneswar, India

## Abstract

The present paper deals with a steady two-dimensional laminar flow of a viscous incompressible electrically conducting fluid over a shrinking sheet in the presence of uniform transverse magnetic field with viscous dissipation. Using suitable similarity transformations the governing partial differential equations are transformed into ordinary differential equations and then solved numerically by fourth-order Runge-Kutta method with shooting technique. Results for velocity and temperature profiles for different values of the governing parameters have been discussed in detail with graphical representation. The numerical evaluation of skin friction and Nusselt number are also given in this paper.

## 1. Introduction

The study of flow and heat transfer over a stretching/shrinking sheet receives considerable attention from many researchers due to its variety of applications in industries such as extrusion of a polymer in a melt spinning process, manufacturing of plastic films, wire drawing, hot rolling, and glass fibre production. Crane [1] first considered the steady laminar boundary layer flow of a Newtonian fluid caused by a linearly stretching flat sheet and found an exact similarity solution in closed analytical form. Chiam [2] investigated the steady two-dimensional stagnation point flow towards a stretching surface in the case when the stretching velocity is identical to the free stream velocity. Hiemenz [3] studied the steady two-dimensional boundary layer flows near the forward stagnation point on an infinite wall using a similarity transformation. Sakiadis [4, 5] reported the flow field analysis where the stretched surface was assumed to move with uniform velocity, and similarity solutions were obtained for the governing equations. The solution is later improved by Howrath [6]. Gupta and Gupta [7] have studied the effect of mass transfer on a stretching sheet with suction or blowing for linear surface velocity subject to uniform temperature. Wang [8] investigated the three-dimensional flow due to the stretching surface. The uniqueness of the solution obtained by Crane [1] was established by McLeod and Rajagopal [9]. Some important contributions in stretching sheet flow were made by Chakrabarti and Gupta [10], Andersson [11], Dirks et al. [12], Jat and Chaudhary [13–17] and Bhattacharyya and Layek [18], and many other authors.

The flow of incompressible fluid due to a shrinking sheet is gaining attention of modern day researchers because of its increasing application to many engineering problems. Lok et al. [19] studied oblique stagnation point flow of Newtonian flow towards a stretching surface. They reported that the position of the stagnation point depends on stretching sheet parameters and the angle of incidence. Hayat et al. [20] reported an analytical solution of MHD flow of a second-grade fluid over a shrinking sheet. Fang and Zhang [21] obtained a closed form analytical solution for steady MHD flow over a porous shrinking sheet subjected to mass suction. Wang [22] studied the stagnation point flow towards a shrinking sheet. Unsteady two-dimensional hydromagnetic flow and heat transfer of an incompressible viscous fluid were investigated by Adhikary and Mishra [23]. Ali et al. [24] investigated the unsteady viscous flow over a shrinking sheet with mass transfer in a rotating fluid. Recently, Bhattacharyya [25] studied the effects of heat source/sink on MHD flow and heat transfer over a shrinking sheet with mass suction.

Realizing the increasing technical application of MHD effects, the present paper studies the effects of magnetic parameter on the boundary layer flow and heat transfer over a porous shrinking sheet with mass suction and viscous dissipation. The physical quantities of interest such as skin friction *C*_*f*_ and Nusselt number Nu are calculated. The numerical results are plotted in some figures and the variations in physical characteristics of the flow dynamics and heat transfer for several parameters involved in the equations are discussed in detail.

## 2. Formulation of the Problem

Consider a steady two-dimensional laminar flow of a viscous incompressible electrically conducting fluid over a permeable shrinking sheet which coincides with the plane *y* = 0 and the flow is confined in the region *y* > 0. The *x*- and *y*-axes are taken along and perpendicular to the sheet, respectively. Two equal and opposite forces are applied along the *x*-axis so that the sheet is stretched keeping the origin fixed. A uniform magnetic field of strength *B*_0_ is assumed to be applied normal to the sheet. The magnetic Reynolds number is taken to be small and therefore the induced magnetic field is neglected. All the fluid properties are assumed to be constant throughout the motion. Under the usual boundary layer approximations the basic governing equations with viscous dissipation are

(1) $$\begin{array}{c} \frac{\partial u}{\partial x}+\frac{\partial v}{\partial y}=0, \end{array}$$

(2) $$\begin{array}{c} u\frac{\partial u}{\partial x}+v\frac{\partial u}{\partial y}=\nu \frac{\partial^{2}u}{\partial y^{2}}-\frac{\sigma B_{0}^{2}u}{\rho }, \end{array}$$

(3) u∂T∂x+v∂T∂y=κρcp∂2T∂y2+Q0ρcp(T−T∞) +μρcp(∂u∂y)2+σB02u2ρcp,

where *u* and *v* are velocity components in *x*- and *y*-directions, respectively, *ν* = *μ*/*ρ* is the kinematic viscosity, *ρ* is the density, *μ* is the coefficient of viscosity, *σ* is the electrical conductivity of the fluid, *T* is the temperature, *T*_*∞*_ is the free stream temperature, *κ* is the thermal conductivity of the fluid, *c*_*p*_ is the specific heat at constant pressure, and *Q*_0_ is the volumetric rate of heat generation or absorption.

The boundary conditions are

(4)  y=0:u=Uw=−cx, v=−vw, T=Tw, y⟶∞:u→0, T⟶T∞,

where *c* > 0, (0 < *c* < 1) is the shrinking constant, *T*_*w*_ is temperature of the sheet, and *v*_*w*_(>0) is a prescribed distribution of wall mass suction through the porous sheet.

The impact of the magnetic parameter *M* on the velocity and temperature is very significant in practical point of view.

**Figure 1:**
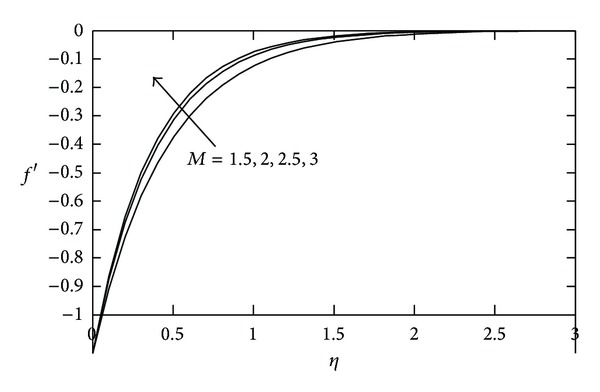
Velocity profile against *η* for various values of *M* when *S* = 2.0.

In Figure 1, the variations in velocity field for several values of *M* are presented. It is observed that the velocity increases with increasing values of *M*. Accordingly, the thickness of the momentum boundary layer decreases. This happens due to the Lorentz force arising from the interaction of magnetic and electric fields during the motion of the electrically conducting fluid.

**Figure 2:**
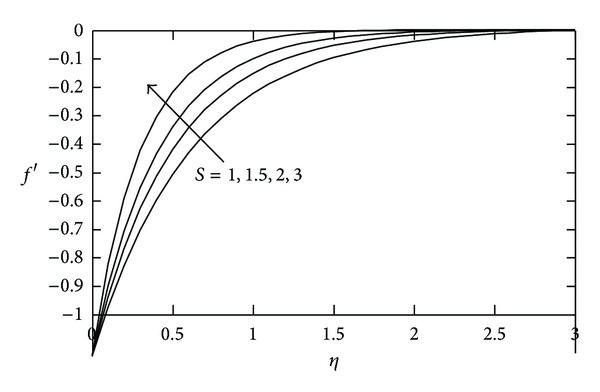
Velocity profile against *η* for various values of *S* when *M* = 2.0.

Figure 2 shows the effects of mass suction parameter *S* on the velocity profile for a fixed value of *M*. It is noted that, for a fixed value of *η*, velocity profile increases as applied suction increases and it makes the boundary layer thickness thinner.

## 3. Mathematical Analysis

The equation of continuity (1) is identically satisfied if we take the stream function *ψ*(*x*, *y*) such that

(5) $$\begin{array}{c} u=\frac{\partial \psi }{\partial y},\;\;v=-\frac{\partial \psi }{\partial x}. \end{array}$$

The momentum and energy equations (2) and (3) can be transformed into the corresponding ordinary differential equations by introducing the following similarity transformations:

(6) $$\begin{array}{c} \psi \left(x,y\right)=\sqrt{c\nu }xf\left(\eta \right),\\ \frac{T-T_{\infty }}{T_{w}-T_{\infty }}=\theta \left(\eta \right), \end{array}$$

where

(7) $$\begin{array}{c} \eta =y\sqrt{\frac{c}{\nu }}. \end{array}$$

The momentum and energy equations (2) and (3) are transformed to

(8) $$\begin{array}{c} f^{\prime \prime \prime }+ff^{\prime \prime }-{f^{\prime }}^{2}-Mf^{\prime }=0,\\ \theta^{\prime \prime }+\text{Pr}\left(f\theta^{\prime }+\lambda \theta \right)+\text{PrEc}{f^{\prime \prime }}^{2}+M\text{PrEc}{f^{\prime }}^{2}=0, \end{array}$$

where *M* = *σ*_*e*_ *B*_0_^2^/*ρc* is the magnetic parameter, Pr = *μc*_*p*_/*κ* is the Prandtl number, *λ* = *Q*_0_/*ρc*_*p*_ *c* is the heat source (*λ* < 0) or sink (*λ* > 0) parameter, and Ec = *U*_*w*_^2^/*c*_*p*_(*T*_*w*_ − *T*_*∞*_) is the Eckert number.

The corresponding boundary conditions are

(9) η=0:f=S, f′=−1, θ=1,η⟶∞:f′⟶0, θ⟶0,

where $S=v_{w}/\sqrt{c\nu }\;\;\left(>0\right)$ is the mass suction parameter.

The physical quantities of interest of the problem are the skin-friction coefficient *C*_*f*_ and the Nusselt number Nu, which can be expressed, respectively, as

(10) Cf=μ(∂u/∂y)y=0(ρU2/2)=2Ref′′(0),Nu=−x(∂T/∂y)y=0(Tw−T∞)=−Re θ′(0),

where *Re* = (*U*_*w*_ *x*)/*ν* is the local Reylolds number.

## 4. Results and Discussion

The set of nonlinear differential equations (8) with boundary conditions (9) were solved numerically using Runge-Kutta fourth-order algorithm with a systematic guessing of shooting technique until the boundary condition at infinity is satisfied. The computations were done by a programme which uses a symbolic and computational computer language, Matlab. Numerical computations are performed for various values of the physical parameters involved, for example, the magnetic parameter *M*, the mass suction parameter *S*, the Prandtl number Pr, the Eckert number Ec, and the heat source/sink parameter *λ*.

In order to assure the accuracy of the applied numerical scheme the computed values of skin-friction are compared with the available results of Muhaimin et al. [26] in Table 1 and have been found to be in very good agreement. The variation of the temperature gradient at the sheet, that is, −*θ*′(0), which is significant in evaluating the rate of heat transfer from the sheet, is presented in Tables 2 and 3.

**Figure 3:**
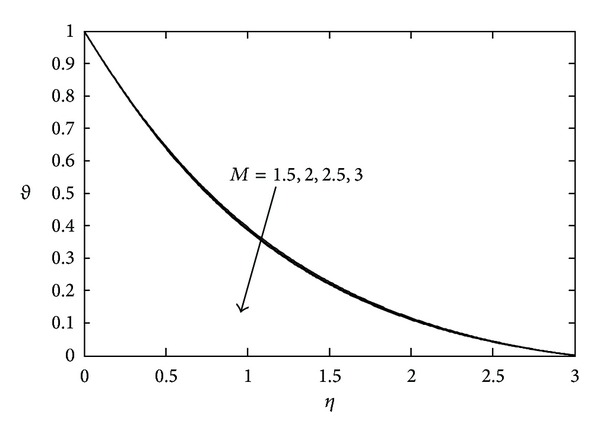
Temperature profile against *η* for various values of *M* when *S* = 2.0, Pr = 0.71, and Ec = 0.1.

**Figure 4:**
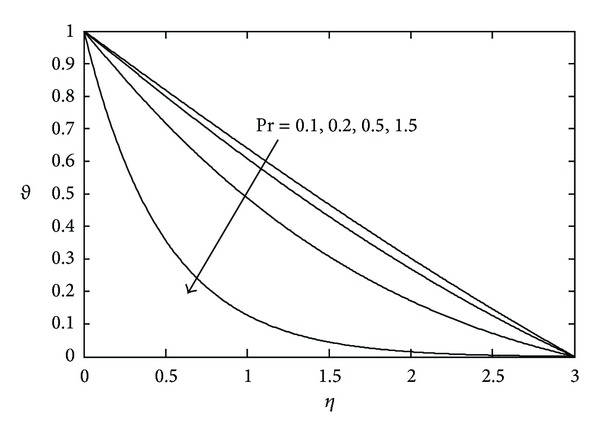
Temperature profile against *η* for various values of Pr when *M* = 2.0, *S* = 2.0, and Ec = 0.1.

**Figure 5:**
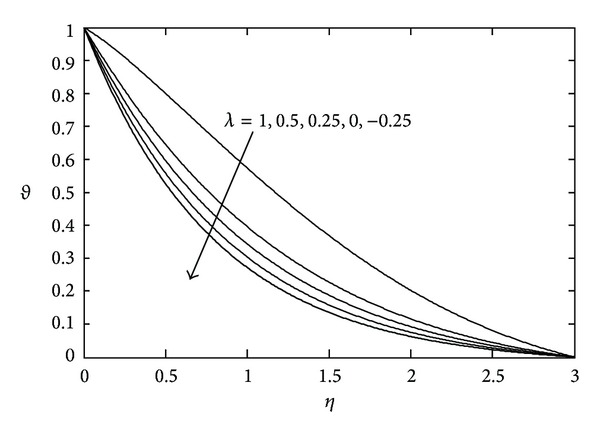
Temperature profile against *η* for various values of *λ* when *M* = 2.0, *S* = 2.0, Pr = 0.7, and Ec = 0.

**Figure 6:**
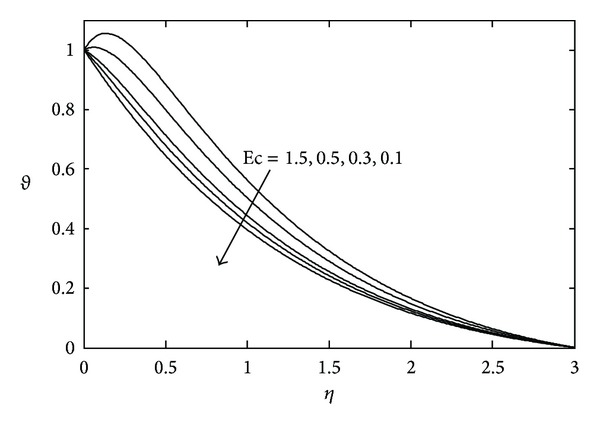
Temperature profile against *η* for various values of Ec when *M* = 2.0, *S* = 2.0, and Pr = 0.7.

Figure 3 shows the effects of magnetic parameter *M* on the temperature distribution. Here it is noticed that the temperature *θ*(*η*) decreases with increasing values of *M*. The temperature field for various values of Prandtl number Pr is represented in Figure 4. With increasing Pr the thermal boundary layer thickness quickly decreases. The temperature field for various values of heat source or sink parameter *λ* is exhibited in Figure 5. It is noted that, for a fixed value of *η*, temperature *θ*(*η*) decreases as *λ* decreases.  Figure 6 shows the effects of Eckert number Ec on temperature *θ*(*η*). It is observed that, for any fixed value of *η*, temperature *θ*(*η*) decreases as Ec decreases.

## 5. Conclusion

The effects of magnetic parameter *M* on the boundary layer flow and heat transfer over a shrinking sheet subject to strong mass suction are studied. The self similar equations are obtained using similarity transformations. The study shows that, due to increase of the magnetic parameter *M* and the mass suction parameter *S*, the momentum boundary layer thickness decreases. Also, the dimensionless temperature profile as well as the thermal boundary layer thickness quickly reduces as Pr increases. Similarly thermal boundary layer thickness decreases as Ec decreases. For some higher values of heat source parameter heat absorption occurs at the sheet. The rate of heat transfer increases with Prandtl number.

## Figures and Tables

**Table 1:** Skin friction coefficient *f*′′(0) for different values of *S* with *M* = 2.0.

*S*	Present study	Muhaimin et al. [26]
2	2.414476	2.414214
3	3.302813	3.302776
4	4.236071	4.236068

**Table 2:** −θ′(0) for various values of Pr when *M* = 2, *S* = 2, and Ec = 0.1.

*λ*	Pr = 0.1	Pr = 0.71
−0.2	0.428487	1.229785
−0.1	0.418625	1.179859
0	0.408646	1.127127
0.1	0.398547	1.071191
0.2	0.388325	1.011564

**Table 3:** −θ′(0) for various values of Ec when *M* = 2, *S* = 2, and Pr = 0.71.

*λ*	Ec = 0.1	Ec = 0.71
−0.2	1.229785	0.50059
−0.1	1.179859	0.443616
0	1.127127	0.383339
0.1	1.071191	0.319285
0.2	1.011564	0.250881
